# Supplementary material for: Health care professionals’ attitudes, behaviours and barriers toward exercise promotion among patients: A systematic review
Source: PLoS One. 2025 Aug 29;20(8):e0330861. doi: 10.1371/journal.pone.0330861 (PMC12396640; doi:10.1371/journal.pone.0330861)
Supplement: S2 Table — (DOCX) [file pone.0330861.s002.docx]

Supporting Information-2

**Supplementary Document 2: Characteristics of included studies**

| **Authors** | **Country** | **Setting** | **Unit** | **Sample Sizes (n=)** | **Participants** | **Design** | **Data collection instrument** |
| --- | --- | --- | --- | --- | --- | --- | --- |
| Al-Ghamdi et al | Saudi Arabia | Primary healthcare | Five districts of Riyadh city | 777 (only reporting health professional data) | 304 physicians, 424 nurses, 31 dieticians, 13 health educators (HE) **Mean age:** 33.06 ± 8.45 years; 60% **female** | Survey | Survey Instrument designed by Douglas 2006 |
| Aldossary et al | Saudi Arabia | Hospitals | 3 key healthcare sectors (government, n = 3; military, n = 3 and private, n = 4) | 744 (not reporting patient data) | Nurses n = 614, doctors n = 130; no age or gender profile provided. | Survey | Primary Care Staff’s Opinions on Promoting Physical Activity sub-scale of the Survey Instrument (Douglas 2006) |
| Avancini et al | Italy | Hospital | Nurses caring for cancer patients | 14 | Nurses; >50% female | Qualitative; focus group | Focus group interview |
| Boltz | USA | Hospital | Nurses working in a unit that serves a population that is composed of at least 50% older adults | 43 (not reporting patient care associate data) | Nurses; no age or gender profile provided. | Qualitative; focus group | Focus group interview |
| Cantwell | Ireland | Hospital and community | Hospital-based oncology healthcare professionals and community-based general practitioners who had previously referred individuals living with and beyond cancer to a community-based exercise programme for cancer survivors | Round 1: 43; Round 2: 48 | Male: Round 1: 35 (15); Round 2: 31 (15); Female: Round 1: 65 (28); Round 2: 69 (33); Physio: Round 1: n=1; Round 2: n=1; Radiation Therapist: Round 1: n=0; Round 2: n=1; Doctor: Round 1: n=23; Round 2: n=19; Nurse: Round 1: n=19; Round 2: n=25; Other: Round 1: n=0; Round 3: n=2 | Delphi study | Questionnaire |
| Chan | Singapore | Hospital | Nurses providing direct bedside nursing care, and with at least six months of experience in their current wards, | 30 | **Age** (years) 28.27 +/-5.99, **Sex:** Male 2 (6.7%); Female 28 (93.3%). | Qualitative; focus group | Focus group interview |
| Cunningham | UK and Ireland | Hospital and community | Healthcare professionals in general practice, physiotherapy, occupational therapy and nursing | 347 | 44 (12.7%) were male and 299 (86.2%) were female. | Survey | Questionnaire |
| De Klein | Netherlands | Hospital | HCPs of two departments: geriatrics and gastroenterology | 9 (not including patient data) | 3 males, 6 females; **mean age** 34 (SD: 10.6); Physio: n=2; Doctor: n=3, Nurse: n=4 | Qualitative | Individual semi-structured interviews |
| Dermody | USA | 2 community-based hospitals | Nurses | 85 | 86% female; | Descriptive correlation survey | Questionnaire: The modified Overall Provider Barriers scale (no ref provided) |
| Doherty-King | USA | 2 Hospitals | Nurses | 26 | No details provided | Qualitative - grounded theory | Interviews |
| Doherty-King | USA | 2 Hospitals | Nurses | 26 | No details provided | Qualitative - grounded theory | Interviews |
| Douglas | UK | Primary healthcare | Nurses (Primary care and Health visitors) | 381for survey; 20 for interviews | 212 PNs and 169 HV. Most respondents were female 205 (99.5%) of PNs and 162 (97%) of HVs, | Mixed methods | Questionnaire and interviews |
| Esposito | USA | Hospital | Nurses | 112 | 93% female, mean age of all respondents: 43 years. | Quailtitative | **Questionnaire: 1.** Beliefs of the benefits of exercise were measured using the Exercise Benefits/Barriers Scale (EBBS). **2.** Exercise behaviour was measured using the physical activity subscale of the HPLP-II (Health-Promoting Lifestyles Profile-II). **3**. Recommendation of exercise to patients was measured using two statements. a. ‘I recommend regular exercise to patients for health promotion’ was used to assess recommendation of exercise for health promotion. b. ‘I recommend exercise to my patients as part of their treatment plan for their condition’, was used to determine recommendation of exercise as part of a treatment plan. |
| Goodman | UK | Primary healthcare | Nurses | 155 | District nurses 169 (33%); Practice nurses 222 (43%); Health visitors 112 (22%); Other nursing specialities 12 (2%) | Quantitative | Questionnaire |
| Hardcastle | International members of seven oncology societies: American Society of Clinical Oncology (ASCO), Clinical Oncology Society of Australia (COSA), Medical Oncology group of Australia (MOGA), Breast Surgeons of Australia and New Zealand (BreastSurgANZ), Canadian Association of Medical Oncologists (CAMO), European Society for Medical Oncology (ESMO), and the European Society for Radiotherapy and Oncology (ESRO) | International medical societies | Ooncologists and oncology health care providers | 123 | medical oncologists (56.8%); surgical oncologists (30.5%); oncology nurses (6.8%), radiation oncologists (4.2%), and others (1.7%). 51.3% female; 61% were aged between 36 and 55 years. | Quantitative | Questionnaire based on the Theory of Planned Behaviour |
| Haussmann | Germany | Hospital | Oncology nurses and physicians working in inpatient and outpatient care in 16 federal states | 699 | 358 physicians; 233 nurses. Mean age: Physicians outpatients: 51.48 ± 8.85, male 54.7%; Physicians inpatients: 39.22 ± 10.28, male 49.8; Oncology nurses: 39.46 ± 10.53, male 18.1. | Quantitative | Questionnaire |
| Jenna | Canada | four urban cancer centres | Health care professionals | 24 | Medical oncologist 7 (29.2); Primary care nurse 5 (20.8); Radiation oncologist 4 (16.7); Surgeon 2 (8.3); Radiation therapist 2 (8.3); General practitioner in oncology 1 (4.2); Nurse practitioner 1 (4.2); Social worker 1 (4.2); Dietician 1 (4.2). 67% female | Qualitative | Interviews |
| Karvinen | USA | Oncology Nursing Society. | Nurses | 274 | female (97.7%); mean age: 48.3 years | Quantitative | Questionnaire |
| Keogh | Australia and New Zealand | the Cancer Nurses Society of Australia (CNSA) and the Cancer Nurses Section of the New Zealand Nurses Organisation (NZNO). | Australasian oncology nurses | 119 | 96% female; 63% ≥45 years | Quantitative | The TPB- and SCT-based online questionnaire. TPB describes how normative beliefs, perceived control, and intentions may influence behavior (Azjen, 1985), and SCT focuses on how behavioral, personal, and environmental factors may influence behavior (Bandura, 1986). Rated barriers on a likert sale. |
| Kime | UK | General practice and community | 15 participants GPs; 3 nurses employed either in a general practice or community | 18 | 11 females; 7 males | Qualitative | Interviews |
| Kleemann | Brazil | 10 Psychosocial Care Units | Nurses and nurse technicians | 75 | Women: 50 (68.5%); Age, years: mean (SD) 37.0 (10.0) | Quantitative | The Portuguese version of The Exercise in Mental Illness Questionnaire (EMIQ). |
| Leemrijse | The Netherlands | Community health care | General practitioners | 340 | 200 males and 140 females; 29 % were younger than 40 years and 30 % were 55 years or older. | Quantitative | Questionnaire |
| Mc Dowell | UK | Community health care | Practice nurses in one county | 220 | All female, with a mean (SD) age of 43.6 (7.9) | Quantitative | Questionnaire |
| Nadler | Canada | Cancer centre | Oncology care providers | 120 | Female 72% (n=87); Median age 48 years (range: 24 to 66). | Quantitative | Questionnaire |
| Din | Wales | Primary healthcare | HCPs of 6 health boards | 46 | 31 GPs, 9 practice nurses, 6 practice managers. No other information provided | Qualitative | Group Interviews |
| Nease | USA | Hospital | four community hospitals focused on non-ICU, non-orthopedic patient care inpatient units. | 219 | 113 nurses, 33 nursing assistants, 42 therapists (physical and occupational therapists and therapy assistants), and 31 providers (physicians and NPs); 82% female | Quantitative | Modification of the survey Instrument designed by Hoyer et al., 2015 |
| Park | Korea | Korean Cancer Association | Oncologists | 123 | 67% male, mean age 43 years | Quantitative | Questionnaire developed by Jones et al (2005) |
| Pearson | USA | Hospital | Nurses who worked in an academic tertiary and quaternary medical centre with a living and deceased donor liver transplant programme | 111 | 81% female; ages N (%) 20–30: 30 (28.0); 31–40: 41 (38.3); 41–50:27 (25.2) | Quantitative | For attitude measurement: The Intent to Mobilise LTR Scale (IMLTRS) developed by the research team-based on the Ajzen’s Theory of Planned Behaviour (Ajzen, 2015). |
| Shirley | Australia | All physical therapists registered in New South Wales | All physical therapists registered in New South Wales | 319 physical therapists | 73% female | Quantitative | Questionnaire based on van der Ploeg et al 2007 |
| Spellman | Australia | Australian cancer clinician membership organisations | Clinicians | 31 | 71% Male; 45% aged 30–40 years | Quantitative | Questionnaire |
| Ungar | Germany | Heidelberg University, the German Cancer Research Center, the National Center for Tumour Diseases, and the Heidelberg University Hospital | Clinicians and nurses | 956 | 552 physicians and 404 nurses; Physicians were on average 46.0 years old (SD = 11.4), and 47.8% were female. Nurses were on average 39.5 years old (SD = 10.5), and 82.4% were female. | Quantitative | Questionnaire based on Ajzen's guidelines |
| Verhaeghe | Belgium | Community health care | Nurses | 17 | No information provided | Qualitative | Focus group interview |
| Williams | UK | Spinal cord units | Physiotherapists | 18 | No information provided | Qualitative | Interviews |
| Wu | Taiwan | 13 long-term care facilities | Nurses | 20 | All female, with a mean age of 40.5 years | Qualitative | Interviews |
