# Supplementary material for: Health care professionals’ attitudes, behaviours and barriers toward exercise promotion among patients: A systematic review
Source: PLoS One. 2025 Aug 29;20(8):e0330861. doi: 10.1371/journal.pone.0330861 (PMC12396640; doi:10.1371/journal.pone.0330861)
Supplement: Attachment — Submitted filename: Reveiw Report.docx [file pone.0330861.s003.docx]

The study topic is of interest and highlights crucial points in the health promotion program. However, there certain limitations that should be addressed:

1. Within the electronic search section specify if there were no filters or limits used,
2. The keyword used in the search process are limited, justify the limited used of keywords for example, physiotherapist or clinician is not included. The keywords exercise or physical activity are also limited as synonyms such as aerobics or movement therapy are not included.
3. Provide operational definitions for HCPs and exercises, as it will simplify the issues concerning selection of keywords.
4. In the study selection section specify that studies in English language were used
5. The initials of the reviewers who did the first screening of the studies are not mentioned in the study selection section
6. On page 8, amend the sentence “… the themes and categories presented under the sections of attitudes, practices, and behaviors.” As “…. attitudes, behaviors and barriers.” As practice was not assessed.
7. Amend the table referring to the CASP assessments to table 3, on page 9.
8. The criteria used in the CASP assessments should be described in the methods section or provided as footnotes in the tables.
9. On page 10, use consistent citation style as per the PLOS One referencing guidelines.
10. I recommend table 2, the characteristics of the studies used to be handled as supplementary file, as it increases the bulkiness of the manuscript without added benefit.
11. How did you manage to avoid bias from the themes already presented in the quantitative questionnaires? As discussed in the limitations, the way questions are phrased or framed can influence how participants respond, leading to response bias and inaccurate measurements of attitudes and themes generated. The same applies to behaviors and barriers, justify.
12. The abbreviations SD and the presented figures are not consistent, if you are referring to SD should be presented in integers not percentages as percentage refers to relative standard deviation (RSD).
13. In the discussion section, page 27, the issue that dancing was not recommended to patients by HCP may be due to the keywords and should not be discussed as a major result.
14. The manuscript needs English language editing.
