# Supplementary material for: Health care professionals’ attitudes, behaviours and barriers toward exercise promotion among patients: A systematic review
Source: PLoS One. 2025 Aug 29;20(8):e0330861. doi: 10.1371/journal.pone.0330861 (PMC12396640; doi:10.1371/journal.pone.0330861)
Supplement: Attachment — Submitted filename: Response Table.docx [file pone.0330861.s005.docx]

Dear Editor,

We would like to thank the reviewer for careful and thorough reading of this manuscript and for the thoughtful comments and constructive suggestions, which help to improve the quality of this manuscript for publication.

We believe that we have carefully addressed all the comments. Should you have any additional requests or questions, please do not hesitate to contact me.

Yours sincerely,

Pinar Avsar

| **Reviewer** | **Comment** | **Answered** | **Page** |
| --- | --- | --- | --- |
| **Review report** | Within the electronic search section specify if there were no filters or limits used, | We have clarified the details regarding the use of filters and limits in the electronic search section of our manuscript. | 7 |
|  | The keyword used in the search process are limited, justify the limited used of keywords for example, physiotherapist or clinician is not included. The keywords exercise or physical activity are also limited as synonyms such as aerobics or movement therapy are not included. | While appreciating the comment of the respected reviewer, the keywords were selected based on the most commonly used terms in the literature to ensure that the search captures a broad range of studies involving healthcare professionals' perspectives on exercise. We intentionally focused on general terms like "healthcare professionals" rather than more specific terms such as "physiotherapist" to avoid narrowing the scope of the search too much. Additionally, while "exercise" and "physical activity" were chosen as umbrella terms to capture a variety of interventions, specific terms like "aerobics" or "movement therapy" were excluded to maintain a manageable scope and to avoid overly fragmenting the search results. However, these terms were considered during the title and abstract screening phase to ensure relevant studies were not missed. |  |
|  | Provide operational definitions for HCPs and exercises, as it will simplify the issues concerning selection of keywords. | To address your concerns, we have added operational definitions for "Healthcare Professionals (HCPs)" and "exercise" to the manuscript. | 8 |
|  | In the study selection section specify that studies in English language were used | Thanks for the comment. It was added. | 7 |
|  | The initials of the reviewers who did the first screening of the studies are not mentioned in the study selection section | Thank you for your feedback. We have added the initials of the reviewers involved in the study selection process to the manuscript for clarity. | 8 |
|  | On page 8, amend the sentence “… the themes and categories presented under the sections of attitudes, practices, and behaviors.” As “…. attitudes, behaviors and barriers.” As practice was not assessed. | Thank you for your suggestion. We have amended the sentence and practice is removed. |  |
|  | Amend the table referring to the CASP assessments to table 3, on page 9. | Thanks for the comment. We amended it. | 9 |
|  | The criteria used in the CASP assessments should be described in the methods section or provided as footnotes in the tables. | Thank you for your suggestion. We have added a "Quality Appraisal" subheading to the methods section, where we describe the criteria used in the CASP assessments, as recommended. | 9 |
|  | On page 10, use consistent citation style as per the PLOS One referencing guidelines. | Thank you for your suggestion. We have revised the citations on page 10 to ensure consistency with the PLOS ONE referencing guidelines. | 10 |
|  | I recommend table 2, the characteristics of the studies used to be handled as supplementary file, as it increases the bulkiness of the manuscript without added benefit. | Thank you for your recommendation. We have moved Table 2, which outlines the characteristics of the studies. This is added as a supplementary file. |  |
|  | How did you manage to avoid bias from the themes already presented in the quantitative questionnaires? As discussed in the limitations, the way questions are phrased or framed can influence how participants respond, leading to response bias and inaccurate measurements of attitudes and themes generated. The same applies to behaviors and barriers, justify. | Thank you for your insightful comment regarding the potential for bias stemming from the themes presented in the quantitative questionnaires. To mitigate this risk, we employed a multi-faceted approach:  Diverse Sources: We ensured that the findings are derived from a variety of sources, including interviews and focus groups, in addition to the quantitative questionnaires. This allowed us to capture a broader range of perspectives and minimize reliance on any single data source.  Thematic Analysis: We conducted a rigorous thematic analysis, where the themes were developed. The emerged themes were reviewed by the research team to ensure the internal validity. This process strived to minimize individual biases and led to a more balanced representation of the data.  Reflexivity: To ensure rigor we acknowledged our own biases and preconceptions during the data analysis process, actively reflecting on how these might influence the interpretation of the findings.  We recognize that response bias can affect the accuracy of measurements related to attitudes, behaviors, and barriers, and we have highlighted these limitations in our discussion. However, we believe that the measures taken have strengthened the validity of our findings. |  |
|  | The abbreviations SD and the presented figures are not consistent, if you are referring to SD should be presented in integers not percentages as percentage refers to relative standard deviation (RSD). | Thank you for your feedback. We have revised the manuscript to ensure consistency in the presentation of the standard deviation (SD), now presenting it as integers rather than percentages. | Whole document |
|  | In the discussion section, page 27, the issue that dancing was not recommended to patients by HCP may be due to the keywords and should not be discussed as a major result. | Thank you for your feedback. We have revised the discussion section. | 28 |
|  | The manuscript needs English language editing. | The manuscript has been edited for English language. | Whole document. |
| **Reviewer #1** | Abstract: Clear and nicely written abstract. However, I think the conclusion section sounds like list of recommendations than summary of the striking data. So please revise. | Thank you for your comment. The conclusion section was revised. | 1 |
|  | Introduction: No need to include the PICO and research question. Probably the primary and secondary desired outcomes can be summarised in the aim part. | Thank you for your suggestion. We have integrated the primary and secondary desired outcomes into the 'Aim' section to provide a more concise overview of the study's focus, as recommended. | 8 |
|  | Methods: I have concerns about the keywords used, for example “behavior” is a key word in the title but not found in the search strategy. Also, you should either use HCPs in general or list all related specialties involved not only physicians and nurses. Someone can argue physiotherapists are more attached to this than nurses, pharmacists ….etc.  It would be useful if you provide the results of the search process on one of the databases as a supplementary (PubMed or Scopus). | Thank you for your valuable feedback. We have updated our search strategy to include the keyword 'behavior' to ensure consistency with the title and the scope of the study.  While we appreciate the insightful comment from the respected reviewer, the decision to use the general term 'healthcare professionals' rather than listing specific specialties such as 'pharmacists' was intentional. This approach aimed to capture a broad range of studies and perspectives without unduly restricting the scope of the search. Using a more general term allowed us to include various healthcare professionals involved in exercise promotion, such as physicians and nurses, ensuring that relevant studies from different disciplines were considered. During the title and abstract screening phase, we carefully evaluated the relevance of each study, including those focusing on specific groups like physiotherapists, pharmacist to ensure that significant contributions were not overlooked. |  |
|  | The manuscript needs language revision for better presentation. | It was added. | Whole document. |
|  | Data analysis section needs extensive revision as I was confused with why meta analysis is essentially mentioned in such type of review with mostly qualitative data (heterogeneity of what? Normally we use this term in studies with quantitative data in nature). How did you do the coding? Is it content analysis or thematic or sentimental ?..... etc | Thank you for your valuable comments. We agree that the term 'meta-analysis' was not appropriate given the qualitative nature of much of the data. As such, we have removed the reference to meta-analysis and clarified that a narrative analysis was used. This narrative approach allowed us to accommodate the diverse methodologies and outcomes of the included studies. We employed a qualitative coding process, which involved creating codes, grouping them into categories, and clustering them into themes. We have updated the manuscript to better explain our analysis process. | 10 |
|  | Results: Where is figure 1? Why did you provide Table 1 with only 3 studies? | Thank you for your comments and for bringing this to our attention. Figure 1 has been provided as a separate file in accordance with the journal's submission requirements. The figure outlines the PRISMA flow diagram, detailing the study selection process, including the number of studies screened, excluded, and included in the review.  Regarding Table 1, we included the details of only three studies to provide a concise illustration of the type of data extracted and analyzed in this review. We believed it was a systematic review requirement to present a summary of excluded studies to enhance transparency and clarify the reasons for exclusion. |  |
|  | In Table 2 you need to add the publication year next to authors and a column for quality assessment. | Thank you for your valuable suggestion regarding the addition of the publication year next to the authors' names and including a column for quality assessment in Table 2. We used EndNote for reference management as required by the journal, and the system's formatting limitations did not allow us to automatically display the publication year directly alongside the authors in the table. |  |
|  | It was very abrupt to see “Data collection techniques” in the results section page 22! Why here ? | Thank you for your observation. The section on 'Data collection techniques' in the results section refers to the methods and approaches used in the included studies, rather than the data collection process for our systematic review. As such, we felt it was most appropriate to include this information in the results section to provide a comprehensive summary of the methodologies used across the studies we reviewed. This placement aims to give readers a better understanding of how data were gathered in the studies included in our analysis. |  |
|  | How did you dealt with data from weaker or incomplete studies? | Thank you for your insightful question. In our systematic review, we adopted a rigorous approach to assess the quality of the included studies using the Critical Appraisal Skills Programme (CASP) tool. For studies identified as weaker or incomplete, we carefully considered their findings in the context of the overall evidence base.  We aimed to extract relevant data while noting any limitations or biases present in these studies. Where applicable, we highlighted the potential impact of these limitations on our findings and discussions. |  |
|  | Discussion:  There are parts where new results were presented for the first time this shouldn't be the case, please revise. The overall discussion needs more depth and critical evaluation. Readers want to see your input for why do you think these were the outcomes of the review. | We have revised the discussion to enhance its depth and critical evaluation. We have also ensured that all presented results align with those previously reported in the literature, avoiding any new findings being introduced. | Discussion section |
| **Reviewer #2** | Reviewer 2's comments overlapped with the review report. Therefore, we have addressed this in our response above. |  |  |
