# Supplementary material for: Health care professionals’ attitudes, behaviours and barriers toward exercise promotion among patients: A systematic review
Source: PLoS One. 2025 Aug 29;20(8):e0330861. doi: 10.1371/journal.pone.0330861 (PMC12396640; doi:10.1371/journal.pone.0330861)
Supplement: Attachment — Submitted filename: Response_Table_auresp_2.docx [file pone.0330861.s006.docx]

Dear Editor,

We would like to thank the reviewer for careful and thorough reading of this manuscript and for the thoughtful comments and constructive suggestions, which help to improve the quality of this manuscript for publication.

We believe that we have carefully addressed all the comments. Should you have any additional requests or questions, please do not hesitate to contact me.

Yours sincerely,

Pinar Avsar

| **Reviewer** | **Comment** | **Answered** | **Page** |
| --- | --- | --- | --- |
| **Additional Editor Comments** | Massive English Editing required. Coherence and readability of the manuscript need an improvement. | We have thoroughly revised the manuscript to enhance its language, clarity, and overall structure. The introduction, discussion, and limitations sections were particularly refined to ensure they are more coherent and easier to follow. | Whole document |
|  | The style of the reference citations should be consistent throughout the manuscript and avoid over-citations where applicable. | Thank you for your feedback. We have revised the manuscript to ensure that the style of reference citations is consistent throughout and have minimized over-citations where applicable. | Whole document |
| **Reviewers** | Please revise the manuscript again . I will put here one paragraph only and comment on the issues in it:  “Geographical location A wide spread of countries are represented in the studies, with the USA having 8 studies(27, 30, 32, 33, 35, 39, 44, 46), the UK having 5 studies (Douglas et al., 2006; Goodman et al., 2011; Kime et al., 2020; Mc Dowell et al., 1997; Williams et al., 2016). Australia, Canada, Germany, The Netherlands, and Saudi Arabia having 2 studies each (17, 24, 25, 29, 38, 41, 43, 47-49). The remaining studies were from Australia and New Zealand (16), Belgium (50), Brazil (14), Ireland (20), Italy (26), Korea (45), Singapore (28), Taiwan (51), The UK and Ireland (15) and Wales (31). One study included people from seven international oncology societies (37).” • Different referencing style. | We have carefully reviewed and edited all references in the manuscript to ensure they adhere to a consistent referencing style format. | Page 11 |
|  | • “Australia, Canada, Germany, The Netherlands, and Saudi Arabia having 2 studies each (17, 24, 25, 29, 38, 41, 43, 47-49)” … one missing reference if each country has 2 papers. | Thank you for identifying the discrepancy in the references. The section has been revised to accurately reflect that Australia, Canada, Germany, The Netherlands, and Saudi Arabia each have two studies, with the correct references now cited as (17, 27, 28, 32, 41, 44, 46, 50-52). | Page 11 |
|  | “The remaining studies were from Australia and New Zealand (16)”.. did you count this as Australian or New Zelandian paper? | Thank you for highlighting this point. The study referenced as (16) was conducted jointly in both Australia and New Zealand, so it was not attributed exclusively to either country. The revised text now clarifies this to avoid any ambiguity. | Page 11 |
|  | Reviewer #2: I have no further comments. However, if the recommendation suggested provided in the first review on the methods used to mitigate the risk of bias stemming from the themes presented in the quantitative questionnaires to be included in the methods section. | Thank you for your valuable suggestion. We have revised the manuscript accordingly and included a detailed subsection under the "Data Analysis" subheading in the Methods section to address this. | 9 |
