# Supplementary material for: Health care professionals’ attitudes, behaviours and barriers toward exercise promotion among patients: A systematic review
Source: PLoS One. 2025 Aug 29;20(8):e0330861. doi: 10.1371/journal.pone.0330861 (PMC12396640; doi:10.1371/journal.pone.0330861)
Supplement: Attachment — Submitted filename: Response to Reviewers.docx [file pone.0330861.s007.docx]

Dear Editor,

We would like to thank the reviewer for careful and thorough reading of this manuscript and for the thoughtful comments and constructive suggestions, which help to improve the quality of this manuscript for publication.

We believe that we have carefully addressed all the comments. Should you have any additional requests or questions, please do not hesitate to contact me.

Yours sincerely,

Pinar Avsar

| **Reviewer** | **Comment** | **Answered** | **Page** |
| --- | --- | --- | --- |
| **Additional Editor Comments** | Massive English editing required. Coherence and readability of the manuscript need improvement.. | Thank you for your feedback. We have revised the manuscript for improved clarity, coherence, and readability to ensure the content is more accessible and aligns with the expectations of the review process. | All document |
|  | Your manuscript does not follow PLOS ONE manuscript preparation guidelines | Thank you for your feedback. We have carefully edited the manuscript to align with the PLOS ONE manuscript preparation guidelines and ensure compliance with all required formatting and structural specifications. | 8 |
| **Journal Requirements** | Please review your reference list to ensure that it is complete and correct. If you have cited papers that have been retracted, please include the rationale for doing so in the manuscript text, or remove these references and replace them with relevant current references. Any changes to the reference list should be mentioned in the rebuttal letter that accompanies your revised manuscript. If you need to cite a retracted article, indicate the article’s retracted status in the References list and also include a citation and full reference for the retraction notice. | We conducted a thorough search using the Retraction Watch Database, PubMed, and the publishers' official websites. None of the cited references in our manuscript have been retracted. |  |
