# Supplementary material for: Health care professionals’ attitudes, behaviours and barriers toward exercise promotion among patients: A systematic review
Source: PLoS One. 2025 Aug 29;20(8):e0330861. doi: 10.1371/journal.pone.0330861 (PMC12396640; doi:10.1371/journal.pone.0330861)
Supplement: Attachment — Submitted filename: Response_to_Reviewers_auresp_4.docx [file pone.0330861.s008.docx]

Dear Editor,

We would like to thank the reviewer for careful and thorough reading of this manuscript and for the thoughtful comments and constructive suggestions, which help to improve the quality of this manuscript for publication.

We believe that we have carefully addressed all the comments. Should you have any additional requests or questions, please do not hesitate to contact me.

Yours sincerely,

Pinar Avsar

| **Reviewer** | **Comment** | **Answered** | **Page** |
| --- | --- | --- | --- |
| **Editor Comments** | - The manuscript does not follow the PLOS ONE manuscript preparation guidelines. - The citation style does not comply with PLOS ONE requirements. - Further English editing is required to improve the coherence and readability of the manuscript. | Thank you for your constructive feedback. In response to your comments, we have thoroughly revised the manuscript to ensure full compliance with the PLOS ONE manuscript preparation guidelines. The citation style has been corrected throughout the text to align with PLOS ONE referencing requirements. Additionally, the manuscript has undergone comprehensive English editing to improve coherence, grammar, and overall readability. We hope that these revisions address your concerns and enhance the clarity and quality of the submission. | All document |
