# Supplementary material for: Health care professionals’ attitudes, behaviours and barriers toward exercise promotion among patients: A systematic review
Source: PLoS One. 2025 Aug 29;20(8):e0330861. doi: 10.1371/journal.pone.0330861 (PMC12396640; doi:10.1371/journal.pone.0330861)
Supplement: Attachment — Submitted filename: Response_to_Reviewers_auresp_5.docx [file pone.0330861.s009.docx]

Dear Editor,

We would like to thank you for providing the opportunity to revise the manuscript. There were three comments. We believe that we have carefully addressed all the comments. Your suggestions have helped us to improve the quality of this manuscript. Should you have any additional requests or questions, please do not hesitate to contact me.

| No | Editorial comments | Response from the Author |
| --- | --- | --- |
| 1 | The manuscript does not follow the PLOS ONE manuscript preparation guidelines | Thanks for the comment. We have reviewed the manuscript preparation guidelines as provided by PLOS ONE. Thorough modifications were made to fit the manuscript into the journal guidelines.  **File format:** The manuscript has been prepared in docx format  **Font:** Standard Arial font has been used throughout the manuscript, font size 12.  **Layout and spacing:** The manuscript is now double-spaced and formatted in a single-column layout.  **Page and line numbers:** Page numbers and continuous line numbers have been included as required.  **Footnotes:** All footnotes have been removed.  **Abbreviations:** Abbreviations are defined upon first use, used consistently. Abbreviations are given at the end of the manuscript. |
| 2 | The citation style does not comply with PLOS ONE requirements. | All references have been formatted according to the Vancouver style, in line with PLOS ONE guidelines.  EndNote was used to manage the references. |
| 3 | Further English editing is required to improve the coherence and readability of the manuscript. | A native language speaker edited the manuscript. We then had the manuscript reviewed by a professional copyeditor. Redundancies were eliminated, sentence restructuring was done as needed, and citations were corrected. This has significantly improved the manuscript. |
